# Gastrodin attenuates rheumatoid arthritis by targeting KAT8 to inhibit the lactylation of H3K9

**DOI:** 10.3389/fphar.2025.1559744

**Published:** 2025-03-27

**Authors:** Yufang Dai, Shunshun Wang, Jiayi Luo, Huiming Yu, Qianqian Du, Shuwan Hou, Minfu Liu, Shan Jiang, Huajiao Xu, Siyi Ye, Muhammad Farrukh Nisar, Haiyan Chen, Jiaqin Wu

**Affiliations:** ^1^ Minda Hospital of Hubei Minzu University, Hubei Provincial Key Laboratory of Occurrence and Intervention of Rheumatic Disease, Enshi, China; ^2^ Hubei Shizhen Laboratory, Wuhan, China; ^3^ School of Pharmacy, Hubei University of Chinese Medicine, Wuhan, China; ^4^ College of Sports and Health, Hubei University of Chinese Medicine, Wuhan, China; ^5^ School of Laboratory Medicine, Hubei University of Chinese Medicine, Wuhan, China; ^6^ Department of Physiology and Biochemistry, Cholistan University of Veterinary and Animal Sciences (CUVAS), Bahawalpur, Pakistan; ^7^ Respiratory Medicine, Wuhan University of Science and Technology Affiliated Geriatric Hospital, Wuhan, China; ^8^ National Innovation and Attracting Talents “111” base, Key Laboratory of Biorheological Science and Technology, Ministry of Education, College of Bioengineering, Chongqing University, Chongqing, China

**Keywords:** rheumatoid arthritis, gastrodin, histone lactylation, KAT8, H3K9, glycolysis

## Abstract

**Introduction:** Rheumatoid arthritis (RA) is a chronic autoimmune disorder characterized by synovial inflammation and joint destruction, with limited therapeutic options. This study investigated the therapeutic potential of gastrodin (GAS), a natural phenolic glycoside derived from Gastrodia elata, in targeting lysine acetyltransferase 8 (KAT8) to suppress histone H3K9 lactylation (H3K9la), a novel post-translational modification linked to inflammatory responses.

**Methods:** The therapeutic effect of GAS on RA was verified by constructing RA models *in vivo* and *in vitro*. Molecular docking, surface plasmon resonance (SPR) assays, overexpression and silencing experiments were used to verify the results.

**Results:** *In vitro* experiments demonstrated that GAS (10–20 μM) significantly inhibited lipopolysaccharide (LPS)-induced expression of pro-inflammatory cytokines (IL-6, MMP1, MMP13) in fibroblast-like synoviocytes (FLS) and THP-1 macrophages by downregulating glycolysis and lactate production. Molecular docking and surface plasmon resonance (SPR) assays confirmed KAT8 as a direct target of GAS, with a dissociation constant (*K*_*D*_) of 413.72 μM. Overexpression and silencing experiments revealed that GAS destabilized KAT8, thereby reducing H3K9la levels. *In vivo*, GAS (20 mg/kg) ameliorated joint swelling and synovial hyperplasia in a Sprague-Dawley rat adjuvant-induced arthritis (AIA) model, correlating with decreased H3K9la and IL-6 expression.

**Discussion:** These findings establish GAS as a promising therapeutic agent for RA by modulating KAT8-mediated histone lactylation, providing new insights into epigenetic regulation of inflammation.

## 1 Introduction

Rheumatoid arthritis (RA) is a chronic autoimmune disorder characterized by synovial inflammation, cartilage destruction, and systemic complications, significantly impairing patients' quality of life (Smolen et al., 2016). Despite advancements in therapeutic strategies, including disease-modifying antirheumatic drugs (DMARDs) and biologics, many patients experience limited efficacy or adverse effects, underscoring the need for novel therapeutic targets (Simon, 2000; Araujo et al., 2016). Recent studies highlight the interplay between metabolic reprogramming and epigenetic modifications in driving RA pathogenesis, particularly the role of glycolysis-derived lactate in promoting inflammatory cascades (Meyer et al., 2023; Payet et al., 2021).

Gastrodin (GAS), a bioactive phenolic glycoside derived from Gastrodia elata, has demonstrated anti-inflammatory, antioxidant, and neuroprotective properties in preclinical models (Xiao et al., 2023). However, its potential in modulating epigenetic pathways remains unexplored (Wang Y. et al., 2024). Lysine acetyltransferase 8 (KAT8), a member of the MYST family, is traditionally recognized for its role in histone acetylation, regulating chromatin structure and gene transcription (Xu and Wan, 2023). Emerging evidence identifies KAT8 as a pan-lactoyltransferase, catalyzing histone lactylation—a novel post-translational modification linking cellular metabolism to inflammatory gene regulation (Xie B. et al., 2024; Zou et al., 2025). Among lactylation sites, histone H3 lysine nine lactylation (H3K9la) has been implicated in macrophage polarization and NF-κB activation, suggesting its critical role in inflammatory diseases (Jaganathan et al., 2014).

In RA, fibroblast-like synoviocytes (FLS) exhibit a hypermetabolic phenotype, relying on glycolysis to sustain proliferation and cytokine production (Nygaard and Firestein, 2020). Lactate, a glycolysis byproduct, serves as both an energy substrate and a signaling molecule, facilitating histone lactylation and perpetuating inflammation (Rabinowitz and Enerback, 2020). While lactate metabolism is increasingly recognized in RA progression, the molecular mechanisms connecting glycolysis, lactylation, and synovial inflammation remain poorly defined. Notably, KAT8-mediated H3K9la has recently been linked to inflammatory gene expression in macrophages, yet its role in RA pathogenesis is unknown (Kuo et al., 2019).

In this study, we found that GAS inhibits synovial cell inflammation by regulating glycolysis using metabolomics assays, and seahorse experiments verified this result. In addition, we found that inhibition of glycolysis effectively suppressed synovial cell inflammation. GAS was further found to inhibit lactate levels and lactylation levels in RA models. Molecular docking and surface plasmon resonance (SPR) experiments verified that GAS regulates the lactic acidification of H3 histone (H3K9la) by affecting the stability of KAT8, which affects synovial cell inflammation. GAS was verified by overexpressing KAT8 (oe-KAT8) and silencing KAT8 (si-KAT8) to regulate H3K9la levels by interfering with KAT8 stability, thereby suppressing inflammation. In this study, we determined that lactylation is involved in the progression of RA and that the level of emulsification is negatively correlated with the expression level of COL2A1. GAS inhibits the inflammation of synoviocytes by suppressing H3K9la levels through downregulation of the stability of KAT8, which leads to the therapeutic effect on RA.

## 2 Materials and methods

### 2.1 Cellular extraction

Synovial tissues (the age range is 43–65, with two females and three males) were obtained from RA patients undergoing joint replacement at Minda Hospital of Hubei Minzu University, with approval from the Institutional Review Board of Hubei Minzu University. All participants provided informed consent. Synovial cell extraction: first, synovial tissue samples cut from arthritic patients with joint replacements were cleaned under sterile conditions. These samples were washed in a sterile phosphate buffer containing antibiotics to remove blood and impurities. The synovial tissue was then cut into small pieces using fine surgical instruments. Next, the tissue pieces were placed in medium containing 10% fetal bovine serum and incubated in an incubator at 37°C with 5% carbon dioxide to allow synovial cells to crawl from the synovial tissue onto the culture flasks to obtain fibroblast-like synoviocytes (FLS). THP-1 monocytes (ATCC TIB-202) were differentiated into macrophages using 100 nM phorbol 12-myristate 13-acetate (PMA).

### 2.2 Cell activity analysis

Cells were seeded and starved in 2% fetal bovine serum (FBS) Dulbecco’s modified Eagle’s medium (DMEM) for 12 h and then incubated with different concentrations of GAS. The cells were mixed with CCK-8 reagent (MCE, HY-K0301) and incubated for 2 h. The absorbance of the samples was recorded at 450 nm at the same time every day for five consecutive days using a 96-microtiter plate apparatus, and the inhibition rate of cell proliferation as well as the IC50 of the GAS in the cells were calculated. These proliferation curves of the cells were plotted for each treatment group. There were 3 times each group of experiments was repeated.

### 2.3 EdU experiment

At a density of 5 × 10^4^ cells/well, 24-well plates were seeded with the identical number of cells. Just 2 hours before to the experiment, the plates were cross-linked to the substrate. The working solution containing 1×EdU was changed out of the medium after 12 h of incubation. After that, the cells were washed with PBS and fixed with 4% PFA fixative. Lastly, the cells were subjected to a permeabilization solution containing Triton X-100 in PBS. They next incubated the mixture for 30 min in the dark at room temperature after adding the click reaction solution. The next step was to add the Hoechst 33,342 solution and let it incubate for 10 min in a dark environment. The cells were rinsed with PBS after incubation. After that, we took pictures of the cells while they were under a fluorescence microscope, and then we divided the total number of cells in the field by the number of EdU fluorescent cells to get the EdU positive rate. There were 3 times each group of experiments was repeated.

### 2.4 RNA extraction and RT-qPCR analysis

The experiment used Trizol reagent, which is manufactured by Invitrogen and located in Carlsbad, CA, United States, to extract total RNA. After that, a StepOne Plus thermocycler (Applied Biosystems, Foster City, CA, United States) was used to conduct a real-time polymerase chain reaction (PCR) with 2× SYBR Green qPCR Master Mix (High ROX) (Bimake, Houston, TX, United States), as instructed by the manufacturer. Starting Points: IL-6(Forward: AGT​GAG​GAA​CAA​GCC​AGA​GC, Reverse: AGC​TGC​GCA​GAA​TGA​GAT​GA), MMP1(Forward: GGT​GAT​GAA​GCA​GCC​CAG​AT, Reverse: GAT​GTG​TTT​GCT​CCC​AGC​GA), MMP13(Forward: CCA​GAC​TTC​ACG​ATG​GCA​TTG, Reverse: GGC​ATC​TCC​TCC​ATA​ATT​TGG​C). Final data were analyzed using the 2^−ΔΔCT^ method.

### 2.5 Western blotting

After 48 h of drug treatment, the cells were separated on ice using normal lysis buffer. After that, a 10% sodium dodecyl sulfate-polyacrylamide gel electrophoresis (SDS-PAGE) was used to separate the proteins, and they were then transferred to a PVDF membrane with a 0.2 μm size. An overnight incubation at 4°C was performed on the membrane with the antibody (Table 1). We treated the membrane with the secondary antibody for 1 h at 37°C after cleaning it with 1x tris buffered saline-Tween (TBST). The Pierce enhanced chemiluminescence (ECL) protein blotting substrate was used to detect the target proteins. Importing the Western blot images that were created was done in ImageJ. Using the ROI manager application, we analyzed and assessed the intensity of all blot bands. Following data collection, GraphPad 9.0 (n = 3) was used for analysis.

**TABLE 1:** Antibody for western blotting.

Antibody	Producer	Cat No
IL-6	Zenbio	500,286
MMP1	Zenbio	383,245
MMP13	Zenbio	381,531
Pan Kla	PTM BIO	PTM-1401RM
H3	Zenbio	381,432
H4	Zenbio	344,323
H3K9la	PTM BIO	PTM-1419RM
H3K14la	PTM BIO	PTM-1414RM
H3K18la	PTM BIO	PTM-1427RM
H3K23la	PTM BIO	PTM-1413RM
H3K27la	PTM BIO	PTM-1428
H3K56la	PTM BIO	PTM-1421RM
H4K5la	PTM BIO	PTM-1407RM
H4K8la	PTM BIO	PTM-1415RM
H4K12la	PTM BIO	PTM-1411RM
H4K16la	PTM BIO	PTM-1417RM
KAT1	Zenbio	R383080
KAT8	Zenbio	R383056

### 2.6 Glycolysis index measurement

Equal numbers of cells from different treatments were introduced into hippocampal XF dishes with 6 × 10^4^ cells per well. Cells were inoculated and incubated in an incubator for 24 h. Matrigel was cross-linked before 2 h. At the end of the incubation, the plates were examined microscopically for healthy, uncontaminated cells. The medium was removed, then washed three times with 500 uL of XF test solution, and finally 500 uL. The plates were then incubated for 60 min in a CO_2_-free environment. The pre-warmed plates were transferred to a hippocampal stage. Glucose (10 mM), oligomycin (1 uM), and 2-DG (50 mM) were added sequentially to the cultures at 100 min were sequentially added to the cultures. Data analysis was performed by Agilent Seahorse Wave software.

### 2.7 Lactate testing

After FLS were blended into a cell suspension, the liquid above was removed and 1 mL of lactate assay buffer was added. The cells were then ground into a powder using sonication in an ice bath for 5 min and then spun at 12,000 rotation per minute for 5 min at 4°C. After separating the liquid, the supernatant was frozen and tested for lactate using a special kit. Lastly, the enzyme marker was subjected to a 30-min preheating period and the detection wavelength was set at 450 nm.

### 2.8 In-cell western

The intracellular western test was carried out in accordance with the protocols provided by the manufacturer. Under aseptic conditions, cells were inserted into black 96-well intracellular WB plates. When the cell coverage was at 70%, the cells were subjected to various treatments for 48 h. Prior to adding CGS-21680, the conditioned medium was withdrawn and incubated at 37°C for 20 min. After that, 4% PFA was used to fix the cells. Each well was treated with 100 μL of LI-COR blocking solution and then shaken at room temperature for 1 h after multiple permeabilization with a 0.1% Triton X-100 PB S solution. Following an overnight incubation of the primary antibodies, dilutions of the secondary antibodies (goat anti-rabbit 800 and goat anti-mouse 680) were introduced and left to incubate in a dark room at room temperature for 1 hour. The last step was to add a 0.1% Tween-20 PBS solution to each well, wash it five times, and then aspirate the fluid. The images were captured using an Odyssey instrument system.

### 2.9 Molecular docking

Structures in GAS format were obtained from the PubChem Compound website, processed with OpenBabel and AutoDock, and then stored. The relevant protein molecules were obtained from the Protein Data Bank (PDB), prepared, and stored using the programs PyMOL and AutoDock. The binding energy was subsequently determined by means of the AutoDock program. The last step was to use PyMOL to visualize and parse GAS and the protein molecule files that went along with it.

### 2.10 Surface plasmon resonance (SPR) experiments

The master mixture was made by dissolving GAS in dimethyl sulfoxide (DMSO). Subsequently, different concentrations of GAS samples were prepared by diluting the master mixture with 5% DMSO flow buffer. The antigen that GAS recognized was coupled to the sensing chip’s surface and injected with GAS. The binding process of GAS-KAT8 was directly monitored through the surface of the chip, and the binding curves allowed for rapid semi-quantification of the kinetic parameters of different antibodies. After sample injection, the pulse appears to have a clear start and end, with continuous buffer flow before and after the pulse. The sensing graph14 shows the binding process and the GAS-KAT complex in its equilibrium state (Wang C. et al., 2024). The amount bound to KAT is a reflection of its concentration and binding affinity; dissociation of the complex is visible upon injection of the sample with buffer in place of KAT.

### 2.11 Animal model

The study protocol was approved by the Animal Care and Use Committee of Hubei University of Chinese Medicine (approval number: 20240221021). SD male rats AIA model were constructed using SPF-grade SD male rats treated with a single intradermal injection of Chevron’s Complete Adjuvant (CFA) 0.1 mL (Biotronik-P2036) in the right hind toe for 10 days. The rats were randomly grouped (n = 5) AIA model group, dexamethasone (DEX)-positive drug group (5 mg/kg), low GAS group (2 mg/kg), and high GAS group (20 mg/kg), and the drug was administered orally once a day for 50 days until the 60th day. The ankle joints and synovial tissues of rats were fixed in 4% PFA, the ankle joints were decalcified, rinsed in PBS, dehydrated in 30% sucrose solution at 4°C for 24 h, embedded in OCT, and cryosectioned at 10 µm thickness. Sections were subsequently stained and analyzed by immunofluorescence staining. Clinical scores for joint swelling were assessed daily using a 0–5 scale (0: no swelling; 5: severe erythema and edema).

### 2.12 Histological analysis

Animal samples underwent fixation in 4% paraformaldehyde for 2 days and subsequent demineralization in 10% EDTA for 2 months. Paraffin embedding followed, yielding 10 μm thick sections. All specimens were analyzed using H&E. Histological scoring was conducted as per prior study protocol. Morphometric assessments utilized ImageJ software. Statistical analysis employed the mean ± standard deviation.

### 2.13 Immunofluorescence staining

Animal sample regions underwent deparaffining, rehydration, fixation, and blocking with 5% bovine serum protein prior to overnight incubation with H3K9la, IL-6, and MMP1 antibodies at 4°C. Following this, HRP-conjugated secondary antibodies were applied for 30 min, followed by staining of the nuclei with DAPI (Sigma Aldrich). Images were captured on a Leica upright microscope and fluorescence intensity was quantified.

### 2.14 Statistical analysis

All statistical analyses were performed using GraphPad Prism 9.0. Data were analyzed using one-way ANOVA followed by Tukey’s *post hoc* test for multiple comparisons. Every value was stated as mean ± standard deviation (SD). ANOVA was used in statistical comparisons to assess variations amongst groups. Considered statistically significant were p-values less than 0.05.

## 3 Results

### 3.1 GAS is not cytotoxic to FLS and THP-1 cells

**FIGURE 1:**
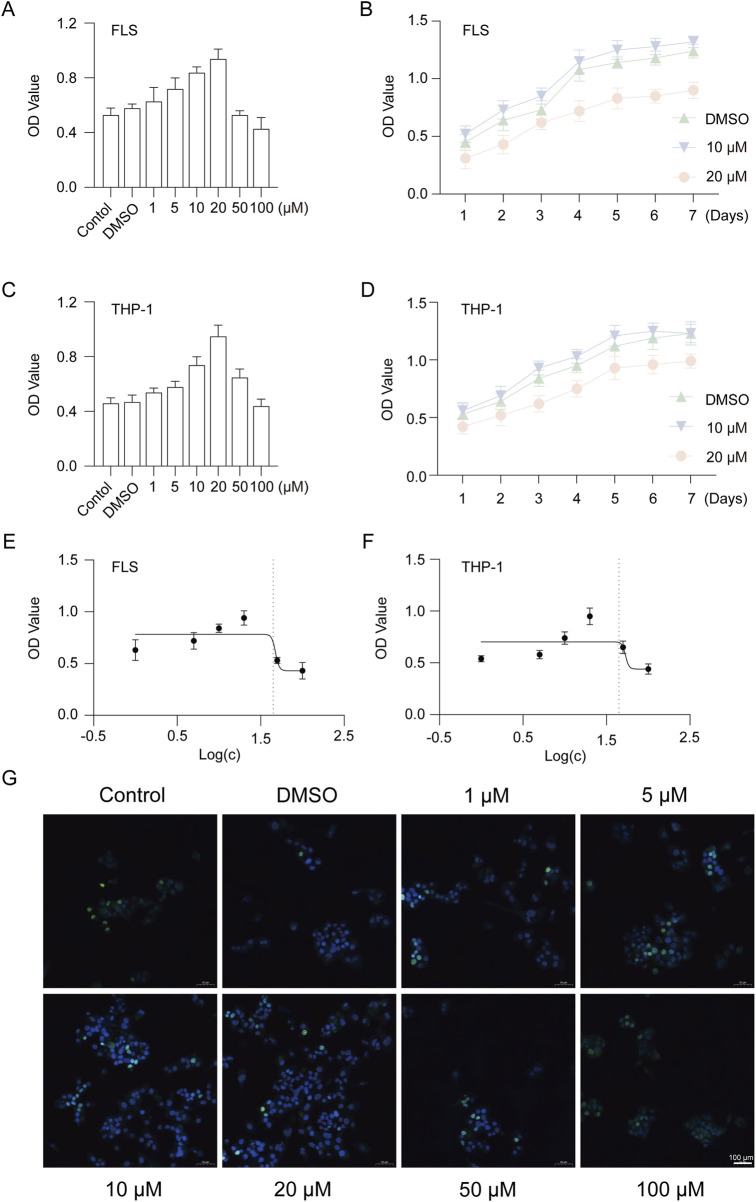
GAS is not cytotoxic to FLS and THP-1 cells. **(A)** CCK-8 assay for cytotoxicity of 1–100 μM GAS on FLS, **(B)** Proliferation of FLS cultured with 10 μM and 20 μM GAS for 1–7 days, **(C)** CCK-8 assay for cytotoxicity of 1–100 μM GAS on THP-1 cells, **(D)** Proliferation of FLS cultured with 10 μM and 20 μM GAS for 1–7 days, **(E, F)** IC50 of GAS-cultured FLS and THP-1 cells, respectively, **(G)** EdU assay for the effect of GAS on FLS proliferation. *p < 0.05; #p < 0.05.

To evaluate the effect of GAS on RA, we cultured cells with different concentrations of GAS to screen the most appropriate culture concentration. The results of *in vitro* cell proliferation assay showed that 10 μM and 20 μM GAS were most suitable for FLS and THP-1 cell proliferation (Figures 1A–D). Cytotoxicity assay showed that the drug inhibited half of the cells (IC50) at concentrations around 47.65 μM and 53.43 μM (Figures 1E,F). EdU proliferation staining assay showed that GAS at 10 μM and 20 μM concentrations had the highest number of proliferating cells in FLS (Figure 1G).

### 3.2 GAS inhibits IL-6, MMP1 and MMP13 expression in FLS and THP-1 cells

**FIGURE 2:**
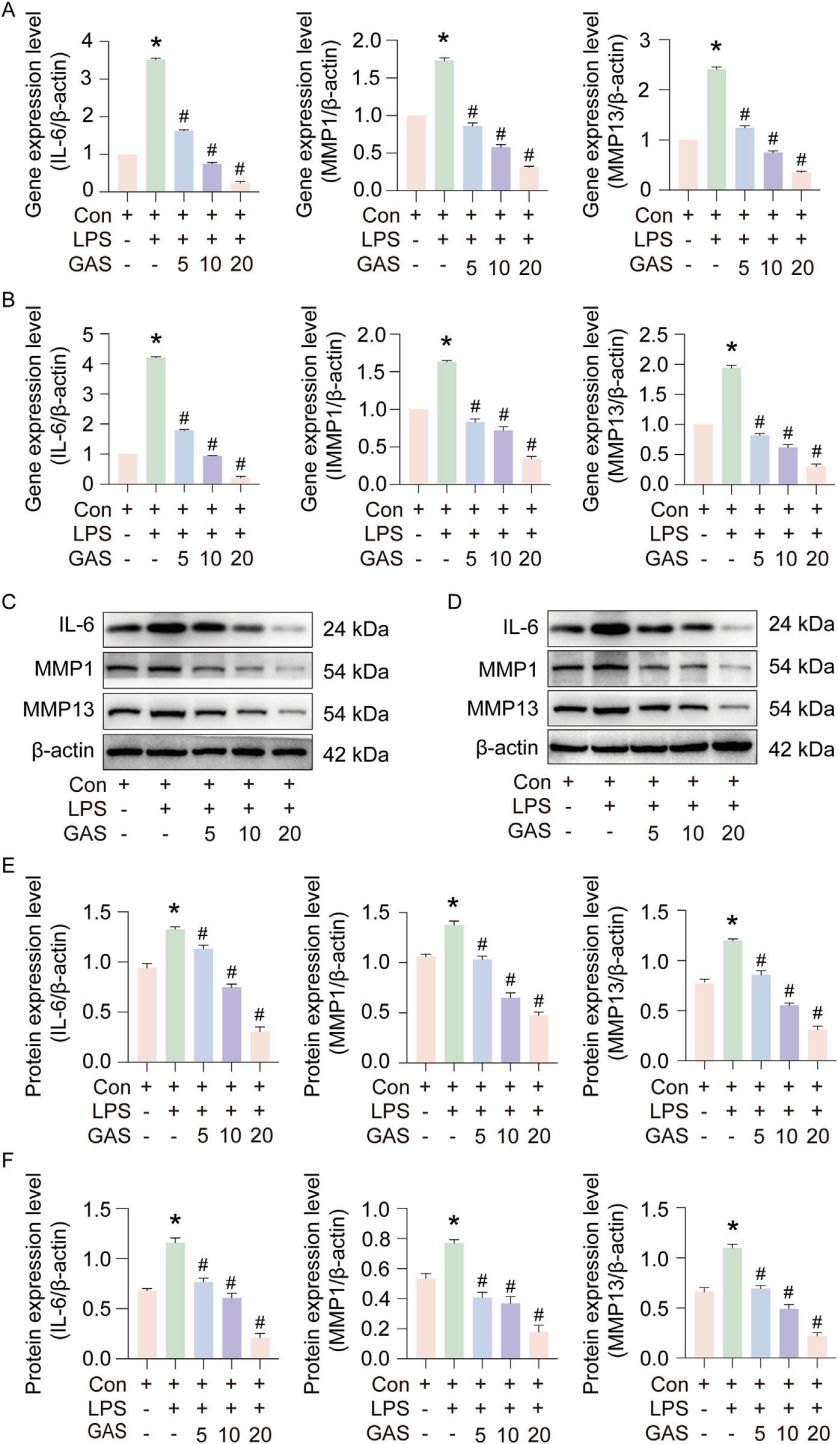
GAS inhibits IL-6, MMP1 and MMP13 expression in FLS and THP-1 cells. **(A)** Effect of GAS on IL-6, MMP1 and MMP13 gene expression in FLS, **(B)** Effect of GAS on IL-6, MMP1 and MMP13 gene expression in THP-1 cells, **(C, E)** Effect of GAS on IL-6, MMP1 and MMP13 protein expression in synoviocytes, **(D, F)** Effect of GAS on IL- 6, MMP1 and MMP13 protein expression in THP-1 cells. *p < 0.05; #p < 0.05. *p < 0.05; #p < 0.05.

In order to observe whether GAS has pharmacological efficacy in inhibiting RA progression, we treated synovial and THP-1 cells with LPS to mimic RA cell models, and cultured the modelled cells with 5, 10 and 20 μM GAS, respectively. Then RT-qPCR and Western blotting were used to detect IL-6, MMP1 and MMP13 gene and protein expression. The results showed that the IL-6, MMP1 and MMP13 gene and protein expression levels were significantly increased after modelling with LPS inflammation, indicating successful modelling, and the IL-6, MMP1 and MMP13 GAS therapy led to a marked reduction in gene and protein expression levels (Figures 2A–F). Based on the data shown in the experiments, GAS may be able to slow down the development of RA.

### 3.3 Inhibition of IL-6, MMP1 and MMP13 expression by GAS is associated with the glycolysis pathway

**FIGURE 3:**
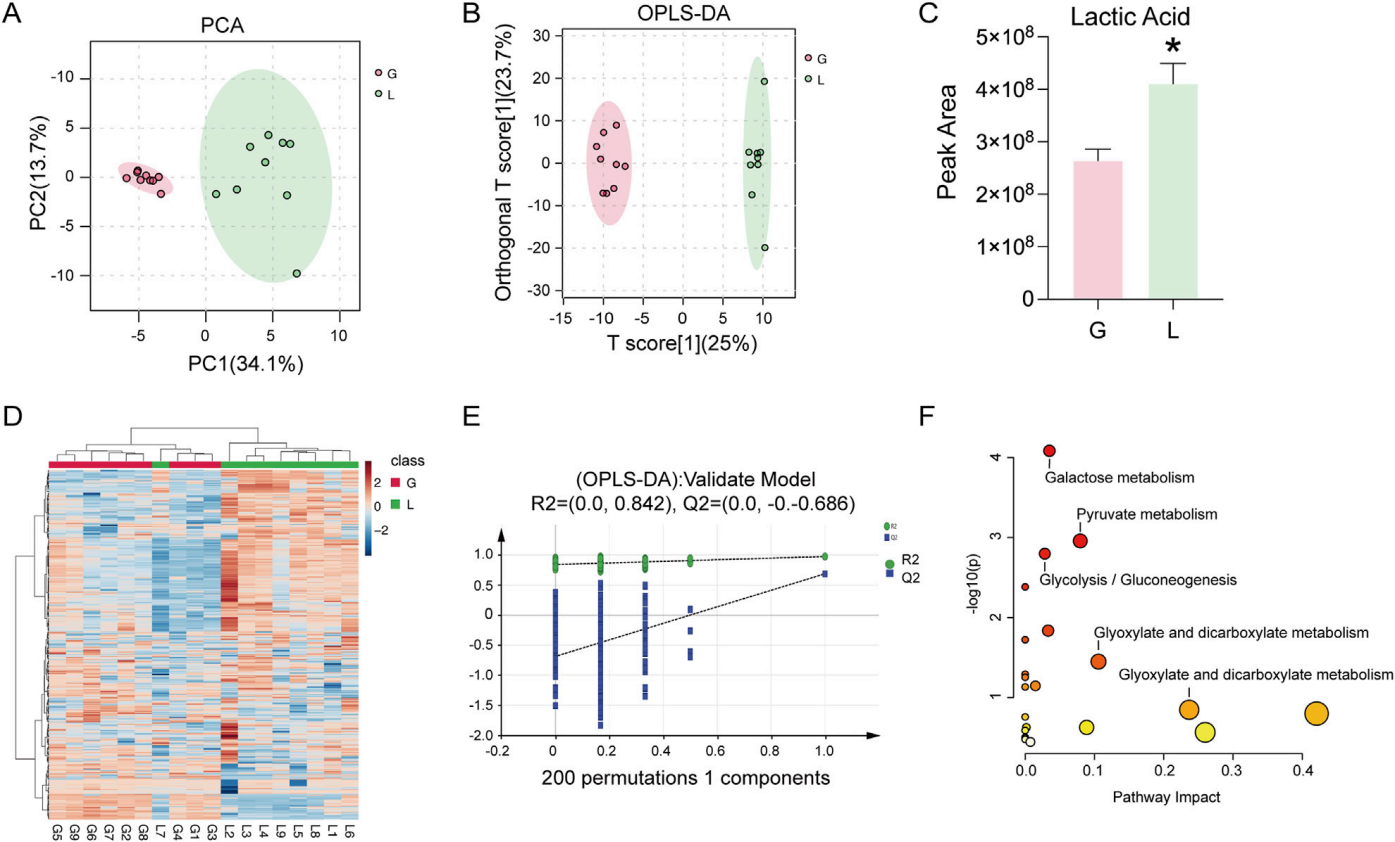
GAS inhibition of IL-6, MMP1 and MMP13 expression correlates with glycolytic pathway. **(A)** PCA score plots of the GTD and IL groups, **(B)** OPLS-DA score plots of the GTD and IL groups showing the clustering of the samples, **(C)** Differential metabolite Lactic Acid content measurements of the GTD and IL groups, **(D)** Metabolite heatmaps showing the differences between the GTD and IL groups, with increased metabolites labelled in red, and decreased metabolites indicated in blue. **(E)** External validation plot of the OPLS-DA model with 200 permutations test, **(F)** KEGG enrichment analysis. *p < 0.05; #p < 0.05. *p < 0.05; #p < 0.05.

To further investigate the molecular mechanism of GAS inhibition of inflammatory mediators, we performed metabolomic analyses, which indicated that glycolysis was contributing to the RA disease inflammatory process. Figure 3A and B show the results of the sample quality evaluation using principal component analysis (PCA) and orthogonal partial least squares discriminant analysis (OPLS-DA). Separate groups were formed from these samples. Analysis of the relative content measurements of the differential metabolites showed a reduction in the metabolite lactate after GAS treatment compared to the LPS group (Figure 3C). Metabolite thermograms showed differences in metabolite concentrations between the LPS combined GAS groups (Figure 3D). The OPLS-DA model was validated using 200 permutation tests, with R2 = (0.0, 0.824) and Q2 = (0.0, −0. −0.686). These results demonstrate that the model is reliable and there is no overfitting (Figure 3E). Metabolic pathway enrichment indicated that the differential metabolites were mainly enriched in galactose metabolism, glycolysis and pyruvate metabolism pathways (Figure 3F). The above metabolomics data suggest that the inhibition of RA progression by GAS may be achieved through the modulation of the glycolysis signalling pathway and the inhibition of lactate metabolism.

### 3.4 GAS suppresses IL-6, MMP1 and MMP13 expression by inhibiting glycolysis

**FIGURE 4:**
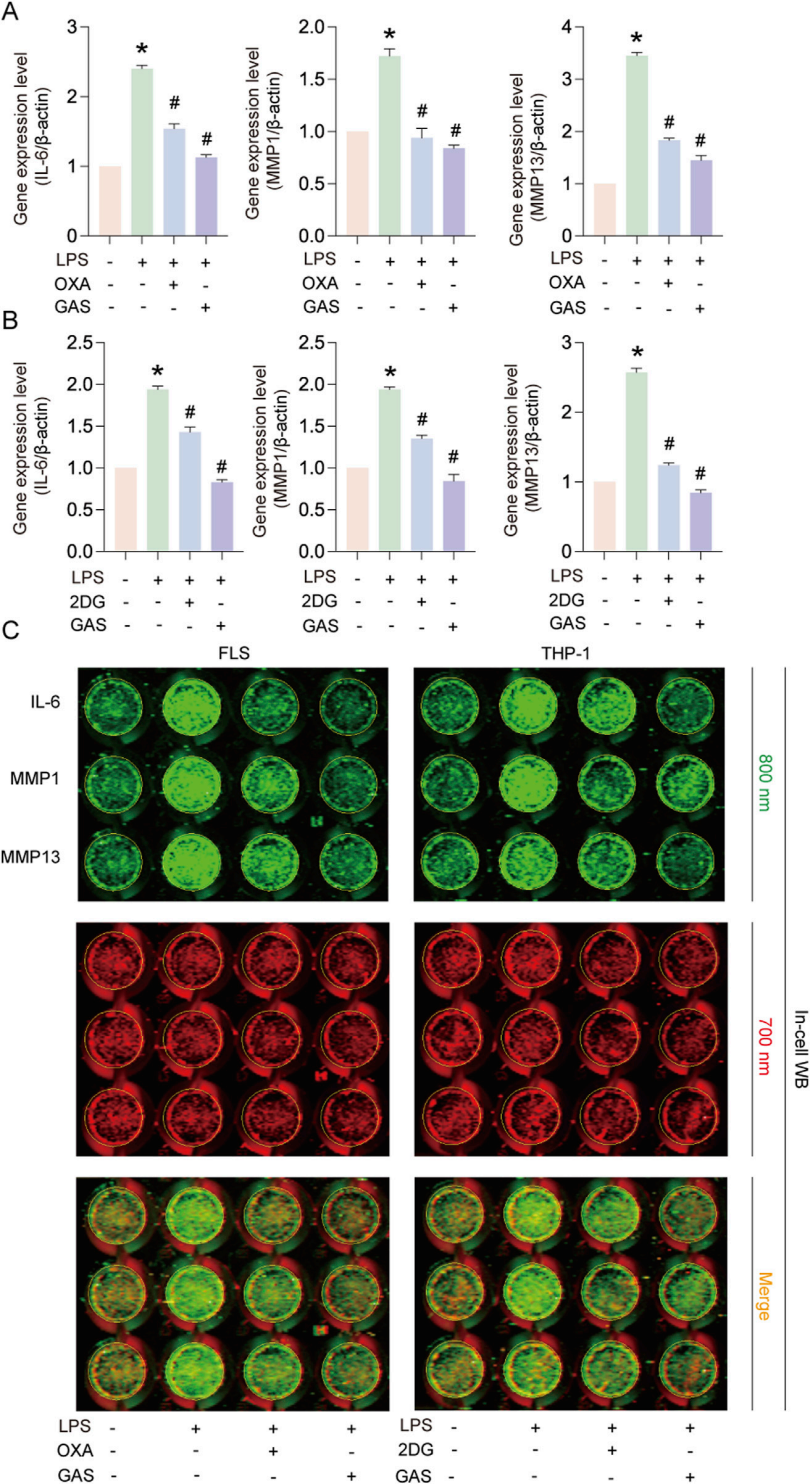
GAS inhibits IL-6, MMP1 and MMP13 expression by inhibiting glycolysis. **(A)** Effects of OXA and GAS on IL-6, MMP1 and MMP13 gene expression in FLS, **(B)** Effects of 2-DG and GAS on IL-6, MMP1 and MMP13 gene expression in THP-1 cells, **(C)** In-cell protein immunoblotting (In-cell WB) to detect the IL-6, MMP1 and MMP13 protein expression levels. *p < 0.05; #p < 0.05.

In order to verify whether GAS inhibits inflammation by modulating the glycolysis pathway, we cultured FLS and THP-1 cells using the glycolysis inhibitors OXA and 2-DG to compare with GAS. Experimental results showed that, similar to GAS’s effects (Figures 4A–C), OXA and 2DG treatments considerably reduced the levels of IL-6, MMP1, and MMP13 protein and mRNA expression generated by LPS. The above experimental results indicated that GAS suppressed IL-6, MMP1 and MMP13 expression by inhibiting glycolysis. The concept that GAS inhibits RA progression possibly by regulating the glycolysis signalling pathway was verified.

### 3.5 GAS inhibits glycolysis and lactate production in FLS

**FIGURE 5:**
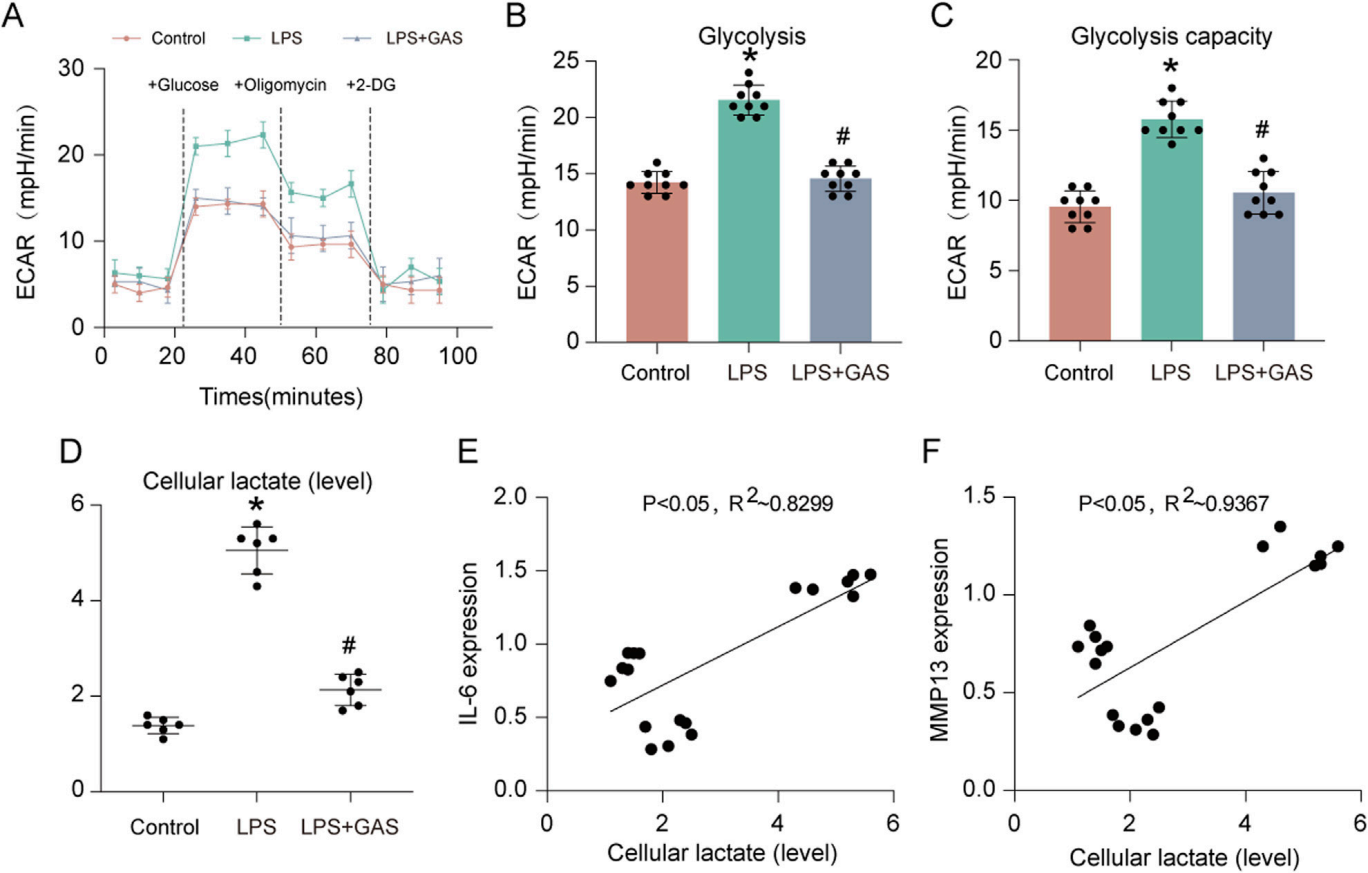
GAS inhibits glycolysis and lactate production in FLS. **(A)** ECAR measurement of FLS, **(B)** statistical analysis of glycolysis, **(C)** statistical analysis of glycolytic capacity, **(D)** lactic acid content of FLS detected by lactic acid assay kit, and **(E–F)** correlation analysis of IL-6 and MMP13 gene expression with lactic acid content of FLS. *p < 0.05; #p < 0.05.

To further verify that GAS could inhibit glycolysis and lactate production in FLS and find how GAS affected the extracellular acidification rate (ECAR) of FLS, we employed Seahorse XF, an extracellular flux detector. While the GAS group was notably lower than the LPS group, the experimental results revealed that the glycolysis level and glycolysis capacity of the LPS-treated group were much higher than those of the control group (Figures 5A–C). To verify that GAS could inhibit the LPS-induced increase in lactate production, we used a lactate assay kit to detect the lactate content of synovial cells. The results of the lactate assay experiments showed that LPS significantly promoted lactate production, whereas GAS significantly inhibited LPS-induced lactate production (Figure 5D). By Pearson correlation analysis, we found that the lactate content in synovial cells was significantly and positively correlated with the expression of IL-6 (P < 0.05, R2 ∼0.8299) and MMP13 (P < 0.05, R2 ∼0.9367) (Figures 5E,F). The above experimental results indicated that GAS could inhibit glycolysis and lactate production in FLS, and the lactate content in FLS was positively correlated with the progression of RA.

### 3.6 Lactate-mediated lactylation of H3 histone H3K9 is a key target of GAS for the treatment of RA

**FIGURE 6:**
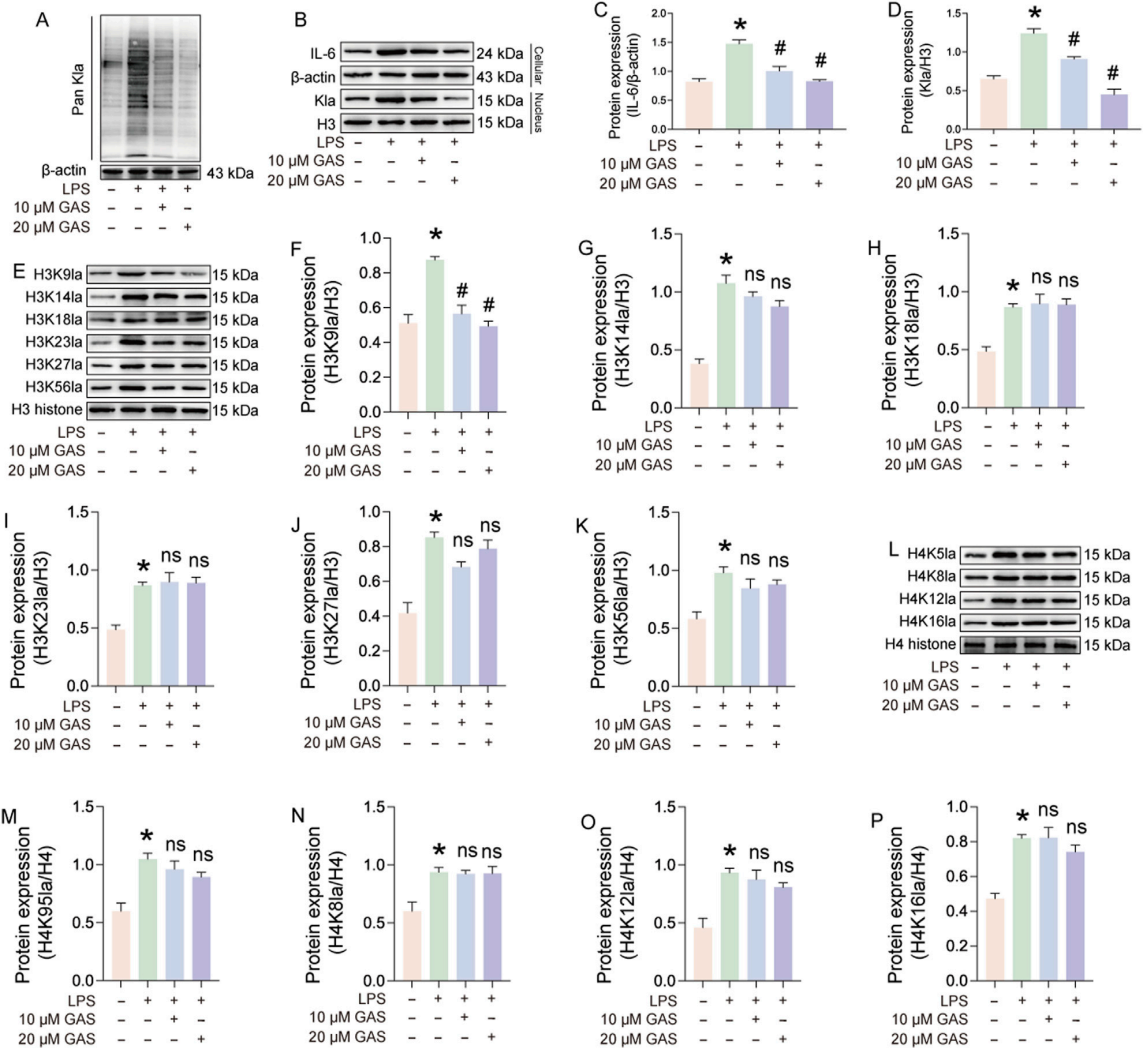
Lactate-mediated lactylation of H3 histone H3K9 is a key target of GAS for the treatment of RA. **(A)** Pan Kla expression levels in LPS and LPS + GAS-treated FLS, **(B–D)** Expression assays of IL-6 in the cytoplasm and Pan Kla in the nucleus of FLS treated with LPS and LPS + GAS, **(E–K)** histone lactylation levels at different sites of H3, **(L–P)** lactylation levels at different sites of histone H4 in FLS. *p < 0.05; #p < 0.05.

Lactate is a substrate for lactylation, and to investigate whether GAS treats RA by inhibiting lactylation, we used Pan Kla antibody to characterize lactylation levels. The results of immunoblotting experiments showed that LPS induction significantly increased the lactylation level of FLS, and the addition of GAS significantly inhibited the lactylation level of FLS, and the inhibitory effect was more pronounced at higher GAS concentrations (Figure 6A). We were concerned that the effect of GAS on histones corresponding to lactylation was even more significant, so we extracted cytosolic and cytoplasmic proteins and detected the expression of IL-6 and lactylation, respectively. The experimental results showed that GAS could effectively reduce the expression levels of IL-6 in the cytoplasm and Kla in the nucleus that were induced to be upregulated by LPS (Figures 6B–D). The above experimental results suggest that GAS may inhibit histone lactylation levels, and to test this hypothesis, we characterized the lactylation expression levels of H3 and H4 histone loci (Figures 6E,L). The experimental results showed that LPS induced upregulation of the lactylation level of histone loci, and interestingly GAS significantly inhibited the LPS-induced upregulation of H3K9la, whereas it did not inhibit the expression of H3K14la, H3K18la, H3K23la, H3K27la, H3K56la, H4K5la, H4K8la, H4K12la, and H4K16la expression were not significantly affected (Figures 6E–P). The above experimental results suggest that lactic acid-mediated lactylation of H3 histone H3K9 is a key target of GAS for the treatment of RA.

### 3.7 GAS targeting of KAT8 reduces its stability to inhibit H3K9la

**FIGURE 7:**
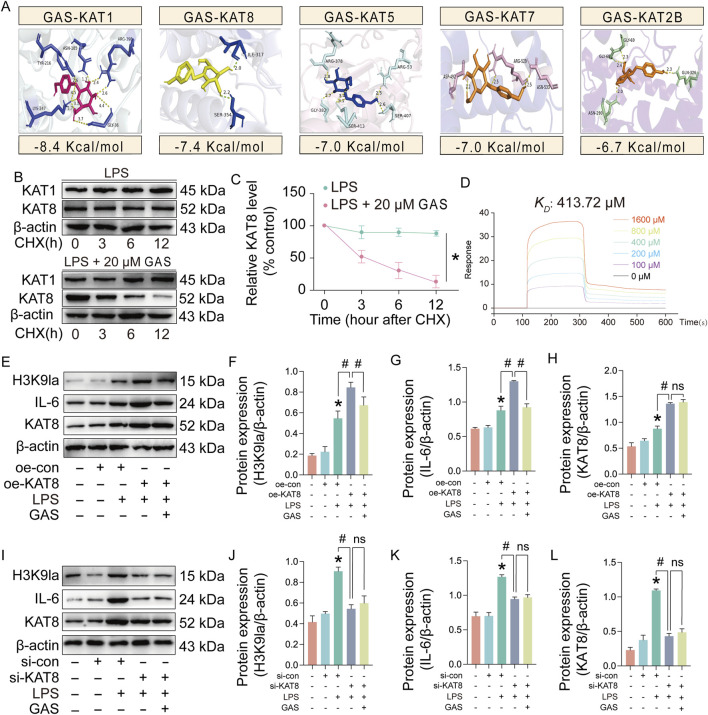
GAS targeting of KAT8 reduces its stability to inhibit H3K9la. **(A)** Molecular docking of GAS and KAT, **(B, C)** Stability ping of KAT1 and KAT8 proteins after LPS and LPS+20 μM GAS treatments, **(D)** Surface plasmon resonance (SPR) analyses of GAS and KAT8, **(E-H)** Protein expression levels of H3K9la, KAT8 and IL-6 in KAT8 overexpressed (oe-KAT8) and GAS-treated synoviocytes, **(I-L)** protein expression levels of H3K9la, KAT8 and IL-6 after KAT8 knockdown (si-KAT8) and GAS-treated synoviocytes. *p < 0.05; #*p* < 0.05.

To further investigate how GAS inhibits the lactylation level of H3K9, we did molecular docking of KAT1, KAT2B, KAT5, KAT7, and KAT8 with GAS, respectively. The molecular docking results showed that GAS had the lowest binding energy to KAT1 and KAT8, suggesting that GAS may bind to them (Figure 7A). The actinomycin ketone (CHX) assay showed that GAS contributes to the destabilization of KAT8 (Figures 7B,C). Surface plasmon resonance (SPR) analysis identified the possibility of GAS binding to KAT8, with a dissociation constant (*K*_*D*_) of 413.72 μM. (Figure 7D). To verify that GAS inhibits H3K9la by binding to KAT8 and contributing to its decreased stability, we overexpressed (oe) and knocked down (si) KAT8. The experimental results revealed that H3K9la and IL-6 expression was much raised in the oe-KAT8-treated group following overexpression of KAT-8 as compared to the LPS group (Figures 7E–G). While adding GAS greatly reduced the expression levels of IL-6 and H3K9la, it had no effect on KAT8 expression level (Figures 7E,H). After knocking down KAT8, the expression of H3K9la and IL-6 was significantly downregulated in the si-KAT8-treated group compared with the LPS group, and there was no significant change in the protein expression of H3K9la and IL-6, nor in the expression of KAT8, after the addition of GAS (Figures 7I–L). The above experimental results suggest that GAS can inhibit the level of H3K9la by interfering with the stability of KAT8, thus inhibiting the expression of IL-6 and exerting a therapeutic effect on RA.

### 3.8 GAS effectively mitigates RA progression in SD rats

**FIGURE 8:**
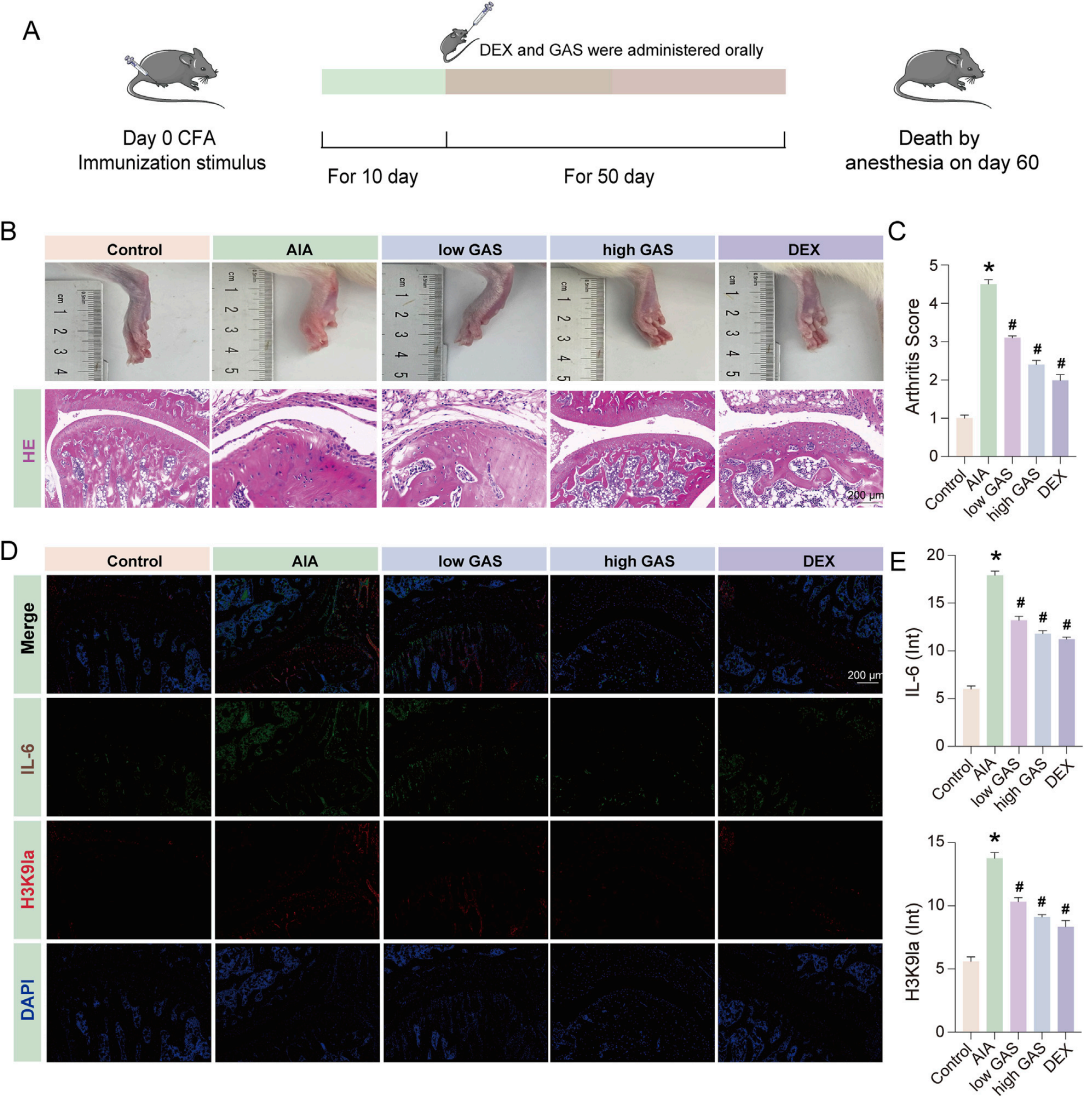
GAS effectively mitigates RA progression in SD rats. **(A)** Schematic diagram of the process of constructing the AIA model in SD rats. **(B)** Histological evaluation in healthy, AIA group, low GAS group, high GAS group and DEX group of ankle joints of SD rat (scale bar: 200 μm). **(C)** Arthritis Score of fig. **(B) (D)** Immunofluorescence staining of IL-6 and H3K9la (scale bar: 200 μm). **(E)** Fluorescence intensity statistics of IL-6 and H3K9la. *p < 0.05; #p < 0.05.

In order to further verify the effect of GAS in the treatment of RA, we carried out animal experiments (Figure 8A). The results of the experiments showed that the modeling group showed symptoms of joint swelling, and the joint swelling improved in the group given GAS treatment (Figure 8B). While synovial hyperplasia and inflammatory cell infiltration were seen in the modeling group shown by H&E staining results, they were lowered in the GAS treatment group (Figures 8B,C). The results of immunofluorescence revealed that the expression levels of lactylation and IL-6 were considerably raised in the modeling group compared with the normal group, and the expression levels of H3K9la and IL-6 were considerably downregulated in the modeling group compared with the GAS treatment group (Figures 8D,E). The above experimental results further verified that GAS has a therapeutic effect on RA from the animal level, and verified that GAS can inhibit H3K9la from the animal level.

## 4 Discussion

RA is a chronic autoimmune disorder for which no cure currently exists (Lee and Weinblatt, 2001). Conventional treatments primarily involve non-steroidal anti-inflammatory drugs (NSAIDs), glucocorticosteroids, and disease-modifying anti-rheumatic drugs, which are often associated with significant side effects and limited efficacy (Kucukdeveci, 2019; Prasad et al., 2023). Traditional Chinese medicine (TCM) has gained increasing attention for its potential in treating chronic diseases like RA due to its multi-target effects, holistic approach, and relatively low incidence of side effects. The study elucidates a novel mechanism by which GAS decreases the protein stability of KAT8 through direct targeting. The inhibition of H3K9 lactylation by GAS ultimately mitigates rheumatoid arthritis and offers novel insights and potential therapeutic targets for its treatment.

As a natural compound, GAS is advantageous due to its wide availability and relatively low incidence of side effects (Dai et al., 2024). This study demonstrates that GAS effectively alleviates rheumatoid arthritis symptoms, thereby providing a theoretical foundation for its clinical application. Over the past decade, extensive research has elucidated the pharmacological properties of gastrodin, revealing its potential as a therapeutic agent for a wide range of diseases (Wan et al., 2021; Liu et al., 2018; Zhang et al., 2023). For instance, GAS has been shown to exert anti-inflammatory effects in models of neuroinflammation and osteoarthritis by inhibiting NF-κB signaling and reducing pro-inflammatory cytokine production (Wang W. et al., 2024; Chen et al., 2018). However, our study is the first to demonstrate that GAS specifically targets KAT8 to inhibit H3K9 lactylation, thereby mitigating RA progression. This novel mechanism highlights the unique therapeutic potential of GAS in RA treatment.

Glycolysis plays a critical role in the pathogenesis of RA, as activated synovial fibroblasts and immune cells rely heavily on glycolytic metabolism to support their pro-inflammatory and proliferative activities (Gan et al., 2024; Xu et al., 2022). Recent studies have explored the therapeutic potential of targeting glycolysis in RA, with inhibitors of key glycolytic enzymes such as hexokinase and pyruvate kinase showing promise in preclinical models (Bustamante et al., 2018; Zuo et al., 2021; Wang Y. H. et al., 2024). Our findings reveal that GAS effectively suppresses glycolysis in RA models, reducing lactate production and restoring metabolic homeostasis. This anti-glycolytic effect of GAS contributes to its anti-inflammatory and anti-arthritic properties.

Lactate, a byproduct of glycolysis, has recently been identified as a key regulator of inflammatory responses through its role in histone lactylation (Dai et al., 2023; Yue et al., 2024). Histone lactylation, particularly at the H3K9 site, has been shown to promote the expression of pro-inflammatory genes, exacerbating inflammation and tissue damage in various diseases (Sun et al., 2021; He et al., 2024). In our study, we observed elevated levels of H3K9 lactylation in RA models, which correlated with increased expression of inflammatory mediators such as IL-6 and MMPs. GAS treatment significantly reduced H3K9 lactylation levels, thereby attenuating inflammation and joint destruction. These findings suggest that H3K9 lactylation is a critical epigenetic mechanism in RA pathogenesis and that targeting this modification could offer a novel therapeutic strategy.

In this study, we identified that GAS specifically targets KAT8, leading to a reduction in its protein stability. This effect is likely mediated through multiple pathways, as evidenced by surface plasmon resonance (SPR) experiments, which confirmed that GAS directly binds to KAT8, thereby influencing its stability. Concurrently, experiments involving the overexpression and knockdown of KAT8 demonstrated that GAS inhibited the expression of H3K9la and inflammatory factors without altering KAT8 expression. These findings further substantiate that GAS directly interacts with KAT8, consequently affecting its stability. KAT8 is receiving increasing attention in biomedical research (Xie B. et al., 2024; Bi et al., 2024; Xie M. et al., 2024). It is now known that KAT8 plays an important role in a variety of biological processes (Huai et al., 2019; Qiu et al., 2023; Zou et al., 2024). Current studies have elucidated that KAT8 plays a pivotal role in the regulation of gene expression by modulating chromatin structure and gene transcription through the acetylation of histones (Radzisheuskaya et al., 2021). Aberrant expression or altered activity of KAT8 has been implicated in the pathogenesis of certain diseases (Xie B. et al., 2024; Chen et al., 2020). Recent studies have identified KAT8 as a pan-lactoyltransferase that facilitates the progression of colorectal cancer (CRC) by modifying the lactylation of the lysine 408 site on the eukaryotic translation elongation factor, eEF1A2, thereby enhancing protein translation efficiency (Qiu et al., 2023).

Our experimental findings demonstrated that in lipopolysaccharide (LPS)-induced FLS and a Sprague-Dawley (SD) rat model of RA, there was an elevated lactylation level of H3K9. This increase promoted the expression of inflammation-related genes, exacerbating joint inflammation and destruction. In contrast, GAS was found to inhibit the lactylation of H3K9 by diminishing the protein stability of KAT8, subsequently mitigating the progression of RA. These results indicate that KAT8 may play a significant role in the pathogenesis of RA, and that GAS can modulate the lactylation level of H3K9 through its interaction with KAT8, thereby alleviating RA symptoms.

However, this study has certain limitations. Primarily, the research was conducted using cellular and animal models, necessitating further clinical investigations to validate the therapeutic efficacy of GAS in human RA. Secondly, this study provides an initial elucidation of the mechanism by which GAS mitigates rheumatoid arthritis, specifically through the targeting of KAT8 to inhibit the lactylation of H3K9. However, further in-depth investigations are required to fully understand its specific mechanism of action. Finally, the current research focuses exclusively on the lactylation processes involving gastrodin, KAT8, and H3K9. Future studies should explore the roles of additional factors in rheumatoid arthritis and their interrelationships with the lactylation of gastrodin, KAT8, and H3K9.

In conclusion, this study elucidates a novel mechanism through which GAS diminishes the protein stability of KAT8 by directly targeting it, consequently inhibiting the lactylation of H3K9 and ultimately ameliorating rheumatoid arthritis. These findings offer innovative insights and potential therapeutic targets for the treatment of rheumatoid arthritis. However, further research is necessary to validate its clinical efficacy and to explore the underlying mechanisms of action.

## Data Availability

The original contributions presented in the study are included in the article/supplementary material, further inquiries can be directed to the corresponding authors.
